# Reduced Hedonic Tone and Emotion Dysregulation Predict Depressive Symptoms Severity during the COVID-19 Outbreak: An Observational Study on the Italian General Population

**DOI:** 10.3390/ijerph18010255

**Published:** 2020-12-31

**Authors:** Lorenzo Moccia, Delfina Janiri, Giulia Giuseppin, Benedetta Agrifoglio, Laura Monti, Marianna Mazza, Emanuele Caroppo, Andrea Fiorillo, Gabriele Sani, Marco Di Nicola, Luigi Janiri

**Affiliations:** 1 Department of Neuroscience, Section of Psychiatry, Università Cattolica del Sacro Cuore, 00168 Rome, Italy; lorenzomoccia27@gmail.com (L.M.); giulia.giuseppin@gmail.com (G.G.); benedetta.agrifoglio@virgilio.it (B.A.); mariannamazza@hotmail.com (M.M.); marcodinicola.md@gmail.com (M.D.N.); luigi_janiri@fastwebnet.it (L.J.); 2 Department of Psychiatry, Fondazione Policlinico Universitario Agostino Gemelli IRCCS, 00168 Rome, Italy; delfina.janiri@gmail.com (D.J.); laura.monti@policlinicogemelli.it (L.M.); 3 Mental Health Department, Local Health Unit ROMA 2, 00173 Rome, Italy; emanuele.caroppo@aslroma2.it; 4 Department of Psychiatry, University of Campania “L. Vanvitelli”, 80138 Naples, Italy; andrea.fiorillo@unicampania.it

**Keywords:** affect regulation, anhedonia, coronavirus, depression, mental health, SARS-CoV-2

## Abstract

The COVID-19 pandemic has spiked stress-related symptoms worldwide. This study aims to assess depressive symptoms related to the early phase of the COVID-19 outbreak among the Italian general population and to analyze anhedonia and emotion dysregulation as potential predictors of depression severity. Through an online questionnaire, we collected sociodemographic and lockdown-related information; depressive symptoms, hedonic tone, and emotion dysregulation were assessed through the Beck Depression Inventory II, the Snaith–Hamilton Pleasure Scale, and the Difficulties in Emotion Regulation Scale, respectively. In our sample (n = 500), 122 individuals (24.4%) reported depressive symptoms during the COVID-19 outbreak. Individuals with and without depression differed in gender (X^2^ = 4.77, df = 1, *p* = 0.02) and age (X^2^ = 15.7, df = 4, *p* = 0.003). Among individuals presenting with depressive symptoms, those reporting close contact with confirmed cases of COVID-19 were at higher risk for severe depression (*p* = 0.026). Reduced hedonic tone (*p* = 0.014) and emotion dysregulation (*p* < 0.001) also predicted depression severity. To the best of our knowledge, these are among the earliest data that focus on the risk for depression among a sizeable sample of the Italian general population during the COVID-19 outbreak. Our results indicate emotion dysregulation and reduced hedonic tone as potential factors predicting COVID-19-related depression severity and provide insight into developing targeted intervention policies.

## 1. Introduction

The COVID-19 pandemic and the resulting isolation have spiked stress-related symptoms worldwide [1]. Italy was the first European Country to face the COVID-19 emergency and to declare a national lockdown [2]. On 9 March 2020, the Italian government imposed a national quarantine, restricting the movement of the population except for necessity, special work permissions, and health conditions. Preventive containment measures during the COVID-19 epidemic, including self-isolation and social distancing, had a strong impact on people’s daily life and adversely affected psychological well-being further.

Individuals experiencing stress symptoms can feel overwhelmed with emotions and can be at higher risk for developing clinical depressive symptoms. However, only mixed evidence is available for whether inter-individual characteristics and demographics may account for determining the psychological response of a population facing massive stressful events [3]. Hence, as the epidemic continues, enhancing our ability to detect possible predictors of the psychological impact during the COVID-19 outbreak is an important focus point. 

“Anhedonia” is derived from the Greek “a-” (without) “hedone” (pleasure, delight) and is described as the inability to gain pleasure from normally pleasurable activities. Pleasure plays a key role in predisposing survival of biological resources and in guaranteeing an essential contribution to the success of adaptive behaviors [4]. Conversely, anhedonia is an obstacle to achieving evolutionary goals, and it has been considered as a core feature of depressive phenotypes [5], insomuch that Klein proposed the existence of a subtype of major depression, referred to as endogenomorphic depression, marked by characterological anhedonic features [6]. Indeed, anhedonia is a required symptom for the diagnosis of a major depressive episode, and evidence suggests that trait anhedonia may represent an important prognostic indicator in individuals suffering from affective disorders [7]. Differences in anhedonia have also been studied on an individual level, suggesting that the subjective hedonic experience originates from brain areas that activate to drive us toward the attainment of primary or secondary human needs [8]. These regions are part of the so-called brain reward system: amygdala, nucleus accumbens, orbitofrontal cortex (OFC), and cingulate cortex. In particular, Zhang et al. [9] suggested that the morphology of the OFC reflects quantitative traits of anhedonia that are continuously distributed throughout the general population and may serve to identify subjects who are at enhanced risk of developing affective disorders.

Emotional responses are multifaceted phenomena that are associated with bodily symptoms, subjective experiences, cognitive changes, and action tendencies, whereas the hedonic marking of emotions is the quality that distinguishes affects from other psychological processes [10]. Research on emotion regulation has highlighted that individuals actively respond and often try to modify their affective states rather than passively experience them. Indeed, emotion regulation broadly refers to the ability to monitor and evaluate emotional experiences, modulate their intensity or duration, and adaptively manage emotional reactions in order to meet situational demands [11]. A substantial body of literature suggests the role of emotion dysregulation in accounting for the onset, overlap, and maintenance of depression [12]. Studies examining emotion regulation in depression have also suggested that depressed individuals exhibit more frequent use of maladaptive strategies, including suppression and rumination, when regulating affects and show difficulties effectively implementing adaptive strategies [13].

The documented connection between epidemics and mental health sequelae dates back more than 100 years ago, when Menninger associated the 1918 Spanish flu pandemic with psychiatric morbidity [14].

Over the past few months, a number of studies reported on the prevalence of depressive symptoms among the general population during the COVID-19 pandemic and identified several potential predictive factors, including age, gender, marital status, education level, occupation, loneliness, having an acquaintance infected with COVID-19, as well as past history of medical disorders [15,16,17]. Conversely, relatively few studies have investigated psychological determinants of depressive symptoms severity during the COVID-19 outbreak [18,19,20,21].

In light of these observations, we aimed at filling this gap by reporting prevalence of depressive symptoms and distribution patterns of hedonic and emotional dysregulation in a sizeable sample of 500 healthy individuals assessed in the early phase of the COVID-19 outbreak in Italy. Further, we sought to identify risk factors predicting depression severity among demographic characteristics, medical and psychopathological variables, and information on lockdown conditions. We hypothesized that reduced hedonic capacity and emotional dysregulation might specifically predict depression severity during the COVID-19 pandemic.

## 2. Materials and Methods

The study was conducted through an on-line survey, starting on 10 April 2020 and ending on 13 April 2020. This timeline was chosen in order to assess participant response during an early phase of the COVID-19 outbreak, after the lockdown in Italy following the governmental decree of 9 March 2020, and the WHO characterization of COVID-19 as a pandemic (11 March 2020). During this period, the total COVID-19 confirmed cases and deaths were 147.577 and 18.849, respectively (Italian Civil Department). The snowball sampling method was employed to recruit participants [22]. In an attempt to ensure an adequately representative sample of the Italian population, an initial subset of invitees (five participants) was selected according to fixed socio-demographic variables, including age groups (18–27, 28–37, 38–47, 48–57, and >57 years old) gender (male, female), occupation (student, employed, unemployed), education level (graduate, undergraduate), and geographic location (northern Italy, central Italy, southern Italy and islands). This subset of participants then forwarded the questionnaire to five referrals whom they considered suitable for the survey. This second subset forwarded the survey in the same way and so on, until data saturation. The survey was anonymous, and confidentiality of information was assured. Participants aged 18–75 years, living in Italy for at least four weeks from February 2020, with adequate command over written and spoken Italian language, and with at least five years of education were eligible for the study. Respondents were excluded if they were non-Italian language speakers, were currently hospitalized, reported a history of mental disorder, and/or could not complete the online survey independently. The study followed the European Survey Research Association (ESRA) guidelines. All participants completed the questionnaire on-line via EUSurvey. The study was approved by the Agostino Gemelli University Hospital Foundation IRCCS-Catholic University of the Sacred Heart of Rome Ethics Committee (approval protocol number ID 3114) and was undertaken in accordance with the Principles of Human Rights, as adopted by the World Medical Association at the 18th WMA General Assembly, Helsinki, Finland, June 1964 and subsequently amended at the 64th WMA General Assembly, Fortaleza, Brazil, October 2013. The provision of electronic informed consent was mandatory in order to start the survey, and anonymity was guaranteed to all participants. The final study sample included 500 subjects from the Italian general population. The number of participants was estimated according to sensitivity analysis conducted on G*Power [23]. Power analysis suggested that an aprioristic sample size of N = 500 would detect with a probability ≥ 0.9 a minimally interesting effect size of δ = 0.2, assuming a two-sided criterion for detection that allows for a maximum Type I error rate of α = 0.05.

A dedicated, self-report questionnaire was adopted to collect main demographic characteristics (age, gender, educational level, occupation, marital status), medical variables (lifetime history of chronic diseases, family history of psychiatric disorders) and information on lockdown conditions (living alone, change in working activities, working on frontline, and having had close contact with confirmed COVID-19 cases; see Supplementary Materials).

To measure the severity of depressive symptoms we adopted the Beck Depression Inventory II (BDI-II) [24], one of the most widely recognized as well as valid and reliable screening tests for depression. Participants must rate their agreement with each statement on a 4-point Likert scale (0 = not at all; 3 = severely) referring to the preceding two weeks. Each of the 21 items of the BDI-II corresponding to a symptom of depression is summed to give a single score. The BDI-II total score ranges from 0 to 63. Consistently with previous validation studies, we adopted a cut-off score of >13 to detect the likelihood of presence of depression [24]. 

Hedonic tone was investigated by the Snaith–Hamilton Pleasure Scale (SHAPS) [25], a 14-item self-report instrument that assesses the ability to feel consummatory pleasure in response to stimuli that typically elicit positive emotion. The SHAPS covers four domains: interests/pastimes, social interaction, sensory experience, and food/drink. Subjects are requested to agree or disagree with a statement for each item on a Likert-type scale (definitely agree, agree, disagree, and definitely disagree). In order to avoid response set, some items are phrased in negative terms. The four available answers are divided into dichotomous categories (agree = 0; disagree = 1). Scores range, therefore, from 0 to 14.

To assess deficits in emotion regulation we used the Difficulties in Emotion Regulation Scale (DERS) [26], a 36-item self-report instrument that targets the individual level of emotion dysregulation. Exploratory factor analysis of the original validation study highlighted a six-factor model for the DERS. The six-factor model is reflected into six subscales yielding a total score: (a) lack of emotional awareness (Awareness; “I am attentive to my feelings”); (b) lack of emotional clarity (Clarity; “I have difficulty making sense out of my feelings”); (c) impulse control difficulties when distressed (Impulse; “When I’m upset, I become out of control”); (d) difficulties engaging in goal-directed behaviors when distressed (Goals; “When I’m upset, I have difficulty getting work done”); (e) unwillingness to accept emotional responses (Non-acceptance; “When I’m upset, I become angry at myself for feeling that way); and (f) lack of access to emotion regulation strategies (Strategies; “When I’m upset, I believe there is nothing I can do to feel better”). Participants rate each item using a 5-point Likert-type scale (1 = almost never; 5 = almost always). The DERS total score ranges from 36 to 180. The higher the DERS total scores, the greater the difficulties in emotion regulation. In prior studies, the DERS has demonstrated convergent validity with other established measures of emotion dysregulation, good test–retest reliability, excellent internal consistency, and adequate predictive validity of several behavioral outcomes associated with emotion dysregulation.

To fit our aims, we compared individuals presenting with and without depression according to BDI-II cut-off scores on the basis of contingency table/χ^2^ for categorical measures, including demographic characteristics, medical variables, information on lockdown conditions, and Student’s *t*-test for hedonic tone and emotional dysregulation. The level of significance was set at *p* < 0.05.

In the group of depressed participants, a multivariate general linear model was conducted to test the effect of all factors of interest on depression severity according to BDI-II total score. Specifically, multiple linear regression was used to predict depression severity on the basis of demographic characteristics, medical variables, information on lockdown conditions, hedonic tone, and emotional dysregulation. Possible multicollinearity between variables of interest was tested through variance inflation factor (VIF) indicators obtained from linear regression analysis. All statistical analyses were performed using SPSS 24.0 for Windows (IBM Corp., Armonk, NY, USA).

## 3. Results

A total of 500 individuals were included in the study. One hundred and twenty-two individuals (24.4%) reported relevant depressive symptoms during the COVID-19 outbreak. Individuals with and without depression differed in gender (χ^2^ = 4.77, df = 1, *p* = 0.02) and age (χ^2^ = 15.7, df = 4, *p* = 0.003). Specifically, among individuals displaying depressive symptoms, most participants were female (n = 83, 68%) and were aged 18–27 (n = 39, 32%). Subjects presenting with depressive symptoms reported reduced hedonic tone (F = 36, df = 1, *p* < 0.001) and higher levels of emotion dysregulation (F = 161, df = 1, *p* < 0.001). The two groups did not differ for medical variables and information on lockdown conditions, including lifetime history of chronic diseases, family history of psychiatric disorders, living alone, change in working activities, working on the frontline, and having had close contact with confirmed cases of COVID-19 (Table 1). 

Among subjects with depressive features, those reporting close contacts with confirmed cases of COVID-19were at higher risk for severe depression. Reduced hedonic tone and emotional dysregulation also specifically predicted depression severity (Table 2). There was no significance of multicollinearity in the model, as indicated by the fact that the VIF of all variables of interest was <2. Regression lines of estimated marginal means depicting the relationship between depression and anhedonia, adjusted for emotional dysregulation, are reported in Figure S2.

## 4. Discussion

To date, the mental health impacts of the COVID-19 outbreak is still under-reported. Individual emotional responses during the massive infectious disease outbreaks are likely to include feelings of extensive fear and uncertainty that, along with social and economic consequences, may eventually cause a dramatic mental health burden [27]. For this reason, we conducted a survey to investigate the occurrence of depressive symptoms among the Italian general population during the early phase of the pandemic. We also went further, by hypothesizing that distinct psychopathological risk factors may conjointly predict depression severity. The results provide additional support for societal concerns of the stressful impact of COVID-19 on mental health.

Our findings indicated, in fact, that nearly 25% of our sample displayed a relevant depressive symptomatology according to BDI-II cutoff scores. Similar rates of depressive symptoms were reported by cross-sectional studies conducted among the general population of worst-hit countries during the initial stage of the pandemic [28,29]. Of note, the majority of individuals in our sample reported no depressive symptomatology. This might be due to the still relatively short exposure to the pandemic or to possible interindividual protective factors promoting mental health [30,31]. Our results also suggested that women and younger individuals were, to a certain degree, more likely to experience significant symptoms of depression in response to the COVID-19 outbreak. These findings may in part be due to the fact that women represent a greater percentage of the workforce that has been negatively affected by the COVID-19 outbreak, including retail, service industry, and healthcare, in addition to biological factors. Similarly, work loss and unpredictability that derived from the COVID-19 pandemic may be particularly stressful among younger age groups [15].

The present findings highlighted that direct exposure to confirmed cases ofCOVID-19 may significantly account for the determination of depression severity. The COVID-19 pandemic may embody a number of negative emotional states and overwhelming stressors. A few of these include loss of employment; deaths of family members, friends, or colleagues; financial insecurity; isolation from others; as well as risk of exposure to contagious individuals. The fact that COVID-19 is human-to-human transmissible, associated with high morbidity, as well as being potentially fatal, may intensify depressive feelings, particularly among those who reported contacts with confirmed cases [32]. In line with our results, a recent population-based study among the community of Wuhan, China identified close contact with individuals with COVID-19 as a risk factor for depression during the first month of lockdown [33].

Decreased emotion regulation abilities as well as anhedonia significantly predicted depression severity in our sample. Sustained negative affects and stressors tend to deplete one’s energy and ability to adaptively cope with situational challenges, which in turn may exacerbate the experience of negative affects, including depressive symptoms [34].

An important issue pertaining to emotion regulation concerns the interindividual variability in experiencing negative or positive affects, as well as the habitual tendency to prefer some regulatory strategies over others to control distressful affects. On the one hand, depression has long been associated with increased levels of negative and stressful affects [35]. On the other hand, one of the key components of emotion dysregulation is the inability to regulate negative emotion and to decrease the duration of negative affect once it arises [36]. Consistently with this conceptual framework, there is evidence linking impaired emotion regulation mechanisms with depressive symptoms, also at a neurobiological level. Indeed, depression has been repeatedly associated with dysfunction in brain regions that are normally implicated in emotion regulation, including prefrontal cortex, (PFC), amygdala, and hippocampus. Intriguingly, these regions have been implicated in the regulation of stress and coping, with the PFC and the hippocampus providing inhibitory control over stress responses, whereas the amygdala has been implicated in potentiating stress-related behaviors [5].

A growing body of evidence suggests that acute stressors may also adversely affect sensitivity to hedonic stimuli [7]. Similarly, anhedonic symptoms have long been conceptualized in terms of blunted response to positive reinforcement, which in turn represents a biological endophenotype of increased depression vulnerability [37]. Taken together, emotion dysregulation and anhedonia may therefore reflect a more general individual incapacity to regulate adaptive responses when facing stressful events, which may ultimately lead to depression. The findings reported here may have practical implications, as the effect of emotion dysregulation and impaired hedonic tone on depressive symptoms is actionable and modifiable through specific interventions on emotion regulation mechanisms. The emerging fields of emotion research and affective neuroscience have, in fact, paved the way for new potential therapeutic venues [38]. This literature points to mutually inhibitory relationships among neural regions implicated in emotion regulation, and a wide network of cortical areas that are involved in downregulating early reactivity to emotionally salient stimuli [11,39].

We understand that the choice of an online survey is not free from methodological risks [40]. However, this was necessary in order to reach a sizeable percentage of the Italian population in a short time during an early phase of the outbreak, when face-to-face contacts are forbidden [41].

Before drawing a study conclusion, we must acknowledge some issues that might mitigate the generalizability of our results. First, the study was carried out throughout four days and lacks longitudinal follow-up. Indeed, the mental health impact of the COVID-19 outbreak on the Italian general population might become more stressful over time. Second, the survey design involved a non-probabilistic sampling method which relied upon the capacity of participants to forward online invitations to others, so that each participant’s suitability may be not controlled for once sample increases in size. Third, people not using network devices, as well as non-Italian language speakers, were excluded. This might represent a selection bias, as there is evidence that COVID-19 disproportionately affects minority groups as well as those living in socially disadvantaged contexts [42]. Fourth, it was not possible to calculate the participation rate, since it is unclear how many individuals received the link for the survey. Finally, the reliability of self-administered questionnaires may be partially biased.

## 5. Conclusions

In conclusion, our result indicate that a relevant percentage of our sample may have experienced relevant depressive symptoms during the early phase of the COVID-19 outbreak and that direct exposure to COVID-19+ confirmed cases, along with emotion dysregulation and anhedonia, may significantly predict depression symptoms severity. It seems fundamental to recognize the potential for mental health consequences of COVID-19 to be large in scale, to identify that these effects can be long-lasting, and to consider preventative action to help mitigate its effects [43]. As the pandemic continues, more work is necessary to clarify risk factors associated with mental health negative outcomes related to the COVID-19 outbreak [44]. Besides, interventions or policies aimed at empowering emotion regulation strategies and stress resilience may have beneficial effects on health and well-being during the COVID-19 pandemic.

## Figures and Tables

**Table 1:** Sociodemographic and psychometric characteristics.

Characteristics (n,%)	Total	No Depressive Symptoms	Depressive Symptoms	χ^2^ or t	df	*p*
**Overall**	500	378 (75.6)	122 (24.4)			
**Age**				15.7	4	**0.003**
18–27	116 (23.2)	77 (20.4)	39 (32.0)			
28–37	129 (25.8)	95 (25.1)	34 (27.9)			
38–47	83 (16.6)	59 (15.6)	24 (19.7)			
48–57	81 (16.2)	70 (18.5)	11 (9.0)			
>57	91 (18.2)	77 (20.4)	14 (11.5)			
**Gender**				4.77	1	**0.029**
Male	202 (40.4)	163 (43.1)	39 (32.0)			
Female	298 (59.6)	215 (56.9)	83 (68.0)			
**Educational level**				1.24	1	0.266
≤Undergraduate	147 (29.4)	116 (30.7)	31 (25.4)			
≥Graduate	353 (70.6)	262 (69.3)	91 (74.6)			
**Occupation**				0.03	2	0.985
Student	72 (14.4)	55 (14.6)	17 (13.9)			
Employed	350 (70.0)	264 (69.8)	86 (70.5)			
Unemployed	78 (15.6)	59 (15.6)	19 (15.6)			
**Marital status**				0.81	1	0.367
Married	181 (36.2)	141 (37.3)	40 (32.8)			
Unmarried	319 (63.8)	237 (62.7)	82 (67.2)			
**Lifetime history of chronic disease**	148 (29.6)	107 (28.3)	41 (33.6)	1.24	1	0.265
**Family history of psychiatric disorders**	67 (13.4)	50 (13.2)	17 (13.9)	0.03	1	0.842
**Living alone**	70 (14.0)	55 (14.6)	15 (12.3)	0.39	1	0.533
**Changes in working activities**	439 (87.8)	331 (87.6)	108 (88.5)	0.08	1	0.779
**Working on frontline**	128 (25.6)	97 (25.7)	31 (25.4)	0.01	1	0.956
**Contact with COVID-19 cases**	65 (13.0)	49 (13.0)	16 (13.1)	0.01	1	0.965
**Psychometric assessment (M ± SD)**						
**SHAPS**	1.2 ± 2.4	0.7 ± 1.6	2.8 ± 3.7	−8.6	1	**<0.001**
**DERS**	75.7 ± 23.1	68.2 ± 16.7	98.8 ± 24.7	−15.4	1	**<0.001**

Significant results in **bold** characters. Abbreviations: M, mean; SD, standard deviation; df, degrees of freedom; χ^2^, chi-squared test; *p*, statistical significance; t, Student’s t; SD standard deviation; BDI-II, Beck Depression Inventory II; SHAPS, Snaith–Hamilton Pleasure Scale; DERS, Difficulties in Emotion Regulation Scale.

**Table 2:** General linear model: Effect of predictors on BDI-II total score.

Predictor	Estimate	SE	95% Confidence Interval		t	*p*
			Lower	Upper		
**Age**	−0.453	0.4370	−1.3195	0.413	−1.037	0.302
**Gender**	−2.243	1.2604	−4.741	0.255	−1.779	0.078
**Educational level**	0.436	1.3793	−2.298	3.170	0.316	0.753
**Occupation**	0.773	1.1109	−1.429	2.975	0.696	0.488
**Marital status**	1.809	1.3260	−0.819	4.438	1.364	0.175
**Lifetime history of chronic disease**	−1.071	1.3192	−3.685	1.544	−0.812	0.419
**Family history of psychiatric disorders**	−0.669	1.6447	−3.929	2.591	−0.407	0.685
**Living alone**	−0.204	1.6858	−3.545	3.138	−0.121	0.904
**Changes in working activities**	−3.006	1.8719	−6.7167	0.704	−1.606	0.111
**Working on frontline**	−1.811	1.4169	−4.6197	0.998	−1.278	0.204
**Contact with COVID-19 cases**	4.052	1.7994	0.4853	7.619	2.252	**0.026**
**SHAPS**	0.393	0.1575	0.0804	0.705	2.492	**0.014**
**DERS**	0.174	0.0234	0.1277	0.221	7.434	**<0.001**

Significant results in **bold** characters. Abbreviations: *p*, statistical significance; SE, size effect; t, t statistic; SHAPS, Snaith–Hamilton Pleasure Scale; DERS, Difficulties in Emotion Regulation Scale.

## Data Availability

The data presented in this study are available on request from the corresponding author.

## Supplementary Materials

The following are available online at https://www.mdpi.com/1660-4601/18/1/255/s1, Questionnaire S1: Survey questionnaire, Figure S2: Regression lines of estimated marginal means depicting the interaction between depression and anhedonia, adjusted for emotional dysregulation.
