# Supplementary material for: Reduced Hedonic Tone and Emotion Dysregulation Predict Depressive Symptoms Severity during the COVID-19 Outbreak: An Observational Study on the Italian General Population
Source: Int J Environ Res Public Health. 2020 Dec 31;18(1):255. doi: 10.3390/ijerph18010255 (PMC7795888; doi:10.3390/ijerph18010255)
Supplement: Supplementary file 1 [file ijerph-18-00255-s001.pdf]

Questionnaire S1.

Parte 1: SCHEDA SOCIO-DEMOGRAFICA

1) Genere:

- Maschio
- Femmina

2) Età (digiti un numero intero): ___________

3) Cittadinanza:

- Italiana
- Straniera

4) Livello di istruzione:

- Licenza elementare
- Licenza media
- Diploma liceale e/o professionale
- Laurea e/o titoli superiori

5) Stato civile:

- Celibe/nubile
- Coniugato/a
- Separato/a o divorziato/a
- Vedovo/a

6) Con chi vive?

- Da solo/a
- Con figli e/o coniuge/partner
- Con altri familiari e/o amici
- Altro

7) Ha figli?

- o Sì
- o No

8) Condizione lavorativa attuale:

- o Lavoratore/rice dipendente a tempo indeterminato
- o Lavoratore/rice dipendente a tempo determinato
- o Lavoratore/rice autonomo/a
- o Assunto mediante Decreto-legge 9 Marzo 2020 (Disposizioni urgenti per il potenziamento del Servizio Sanitario Nazionale in relazione all'emergenza COVID-19)
- o Disoccupato/a o in cerca di prima occupazione
- o Studente/ssa
- o Casalingo/a
- o Pensionato/a

9) Appartiene ad una delle seguenti categorie professionali?

- o Personale medico

(Digiti la sua specializzazione medica): ______________________________

- o Personale infermieristico
- o Altro personale sanitario o parasanitario
- o Psicologo/a
- o Forze armate/Polizia
- o Pubblica Amministrazione
- o Nessuna delle precedenti

10) In merito alla sua salute personale, soffre di patologie mediche croniche e/o assume abitualmente terapie farmacologiche?

- o Sì
- o No

11) Soffre di disturbi psichici (incluse dipendenze da alcol, droghe, gioco d'azzardo) e/o è in trattamento con psicofarmaci?

- Sì
- No

12) Qualcuno fra i suoi familiari di primo grado soffre di disturbi psichici (inclusi dipendenze da alcol, droghe, gioco d'azzardo) e/o è in trattamento con psicofarmaci?

- Sì
- No

Parte 2: SCHEDA INFORMATIVA RELATIVA ALLA PANDEMIA DA COVID-19

13) Città o comune italiano in cui ha vissuto/lavorato prevalentemente nelle ultime 4 settimane (digiti il nome della città o comune): ______________________________________

14) Ha vissuto nella stessa città o comune dei suoi familiari nelle ultime 4 settimane?

- Sì
- No

15) In seguito alla diffusione del Nuovo Coronavirus 2019 (Covid-19) sul territorio italiano:

- Ha sviluppato sintomi riconducibili al Coronavirus, ha eseguito il tampone ed è risultato positivo
- Ha sviluppato sintomi riconducibili al Coronavirus, ha eseguito il tampone ed è risultato negativo
- Ha sviluppato sintomi riconducibili al Coronavirus ma non ha eseguito il tampone
- Ha avuto contatti diretti con soggetti risultati positivi
- Non ha avuto contatti diretti con soggetti risultati positivi

16) Ha effettuato un periodo di quarantena o isolamento precauzionale in seguito a contagio o esposizione sospetta?

- No
- 1-3 giorni
- 4-7 giorni
- 8-14 giorni
- Superiore a 14 giorni

17) È stato attivamente coinvolto nella gestione dell’emergenza sanitaria da Covid-19?

- Sì
- No

18) È venuto a diretto contatto con persone decedute per Covid-19?

- Sì
- No

19) Come si sono modificate le sue abitudini di studio/lavorative nelle ultime 4 settimane, in relazione alle nuove disposizioni di Governo?

- Nessun cambiamento
- È stato necessario ridurre o interrompere totalmente l'attività
- È stato necessario intensificare l'attività
- Ha fatto maggior ricorso allo “smart working” o prestazioni/lezioni telematiche
- Altro

20) Ha effettuato visite di controllo presso il proprio medico di famiglia, pediatra o guardia medica nelle ultime 4 settimane in relazione al Covid-19?

- Nessuna
- 1-3
- 3-6
- Più di 6

21) Qualora sia affetto da un disturbo psichico (incluse dipendenze da alcol, droghe, gioco d’azzardo), ha effettuato una consultazione urgente (Psichiatra/Psicologo curanti) nelle ultime 4 settimane in relazione al Covid-19?

- Non sono affetto da alcun disturbo psichico
- Nessuna
- 1-3
- 3-6
- Più di 6

22) Ha effettuato accessi nei dipartimenti di emergenza (Pronto Soccorso, DEA) nelle ultime 4 settimane in relazione al Covid-19?

- ○ Nessuna
- ○ 1-3
- ○ 3-6
- ○ Più di 6

Figure S2.

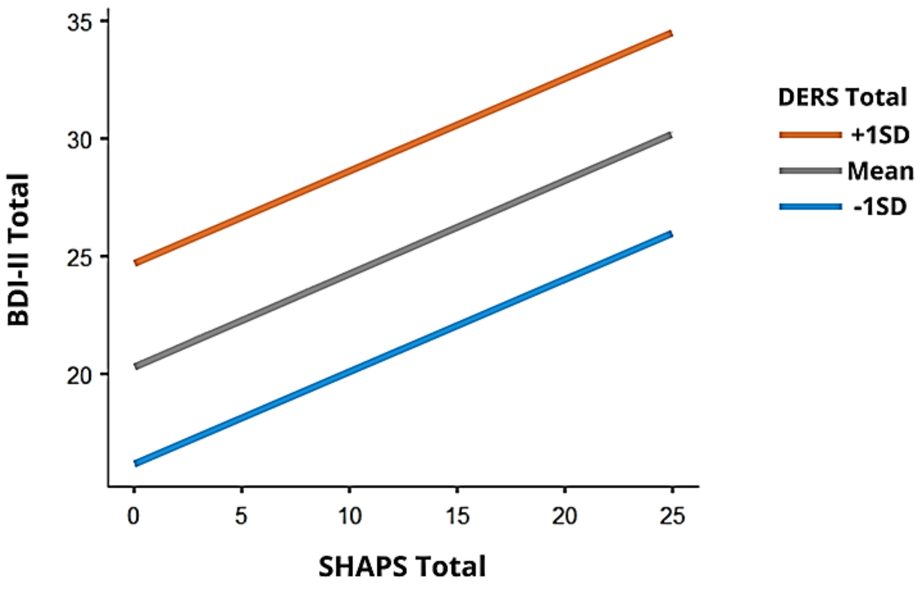

Figure S2. Regression lines of estimated marginal means depicting the interaction between depression and anhedonia, adjusted for emotional dysregulation. Abbreviations: BDI-II, Beck Depression Inventory II; SHAPS, Snaith–Hamilton Pleasure Scale; DERS, Difficulties in Emotion Regulation Scale; SD, standard deviation.
